# The Early Outcomes of Nurse Case Management in Patients with Acute Ischemic Stroke Treated with Intravenous Recombinant Tissue Plasminogen Activator: A Prospective Randomized Controlled Trial

**DOI:** 10.1155/2018/1717843

**Published:** 2018-06-07

**Authors:** Urai Kummarg, Siriorn Sindhu, Sombat Muengtaweepongsa

**Affiliations:** ^1^Faculty of Medicine, Thammasat University, Pathum Thani 12120, Thailand; ^2^Faculty of Nursing, Mahidol University, Bangkok 10700, Thailand

## Abstract

**Background:**

Intravenous recombinant tissue plasminogen activator (i.v. rt-PA) is the milestone treatment for patients with acute ischemic stroke. Stroke Fast Track (SFT) facilitates time reduction, guarantees safety, and promotes good clinical outcomes in i.v. rt-PA treatment. Nursing case management is a healthcare service providing clinical benefits in many specific diseases. The knowledge about the efficacy of a nurse case management for Stroke Fast Track is limited. We aim to study the effect of nurse case management on clinical outcomes in patients with acute ischemic stroke involving intravenous recombinant tissue plasminogen activator (i.v. rt-PA) treatment.

**Methods:**

Seventy-six patients with acute ischemic stroke who received i.v. rt-PA treatment under Stroke Fast Track protocol of Thammasat University Hospital were randomized into two groups. One group was assigned to get standard care (control) while another group was assigned to get standard care under a nurse case management. The National Institute of Health Stroke Scale (NIHSS) at 24 hours after treatment between the control and the experimental groups was evaluated.

**Results:**

Time from triage to treatment in the experimental group was significantly faster than in the control group (mean = 39.02 and 59.37 minutes, respectively; p=.001). The NIHSS at 24 hours after treatment in the nurse case management group was significantly improved as compared to the control group (p=.001). No symptomatic intracranial hemorrhage (sICH) was detected at 24 hours after onset in both groups.

**Conclusion:**

The nurse case management should provide some benefits in the acute stroke system. Although the early benefit is demonstrated in our study, further studies are needed to ensure the long-term benefit and confirm its profit in patients with acute ischemic stroke.

## 1. Introduction

Stroke is the important cause of adult disability particularly in the elderly and remains the third most common cause of death in the developing world [1] as well as in Thailand [2–4]. The prevalence of stroke is one percent in Thai people aged more than 30 years [5]. Intravenous recombinant tissue plasminogen activator (i.v. rt-PA) is an essential treatment for patients with acute ischemic stroke [6, 7]. The i.v. rt-PA treatment is a time constraint therapy; therefore, Stroke Chain of Survival is the mandatory linkage of systems to ensure treatment reach for stroke patients [8]. The 8 steps (8Ds) in Stroke Chain of Survival consist of out-of-hospital part: detection, dispatch and delivery, and so-called Stroke Fast Track, and in-hospital part: door, data, decision, drug, and disposition [9]. Stroke Fast Track with referral network facilitates time reduction, guarantees safety, and promotes good clinical outcomes in i.v. rt-PA treatment [10].

Nursing case management is a healthcare service provided for defined groups of patients [11]. It shows clinical benefits in many specific diseases. For example, nurse case management helps long-term cholesterol control in patients with coronary heart disease [12]. In patients with ischemic stroke, nurse case management improves risk reduction outcomes in a stroke prevention clinic [13]. Moreover, nurse case management in stroke unit leads to good outcomes in relation to interdisciplinary utilization, timeliness of referrals, patient education, discharge dispositions, home safety assessments, next-site-of-care communications, the length of hospital stay, and patient satisfaction [14]. However, little is known about the efficacy of a nurse case management for Stroke Fast Track. The roles of nurse case manager in Stroke Fast Track include knowledge and practice in triage, neurological examination, especially National Institute of Health Stroke Scale (NIHSS) assessment, and the interpretation of the computerized tomography of the brain. We aim to study the effect of nurse case management on clinical outcomes in patients with acute ischemic stroke involving Stroke Fast Track.

## 2. Materials and Methods

### 2.1. Study Design

This is a prospective, single-blinded, randomized controlled study.

### 2.2. Setting and Sample

Thammasat University Hospital (TUH) is a 460-bed hospital in Thailand with integrated acute stroke referral network [10]. There are approximately 1,000 patients who participated in Stroke Fast Track (SFT) protocol with 400 of them receiving i.v. rt-PA every year. Patients who received i.v. rt-PA treatment after March 2014 with age between 35 and 70 years were randomized into two groups. One group was assigned to get standard care (control) while another group was assigned to get standard care under a nurse case management. Sample size is estimated on the basis of power analysis with the level of significance at 0.05, the effect size at 0.60, and the power of test at 0.08. From Lipsey (1990) [15], the sample size is 35 cases for control group and 35 cases for experimental group. For prevention of the error from dropout, the sample size should be added 10%. The sample size in this study is 38 for each group. The enrollment was stopped when the eligible patients reached 76.

### 2.3. Ethical Consideration

The study was approved by the Mahidol University Institutional Review Board (MU-IRB) and the Ethics Committee of Thammasat University Hospital. All patients have a right to refuse this study at any time. The researcher does not have to count these patients in the study nor use their data in the study.

### 2.4. Measurements

#### 2.4.1. Control Group

**Figure 1:**
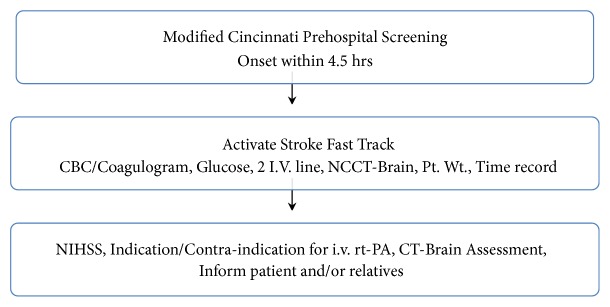
The flow chart of Stroke Fast Track.

The standard care was under responsibility of emergency nurses and physicians. Emergency nurses gave nursing care following the job description as a nursing care team. The nursing care team consisted of one registered nurse who became in charge and one practical nurse who became a member. A triage nurse at the front desk was responsible for an initial assessment and screening. When patients with potential stroke came in, the triage nurse activated SFT protocol and alerted the nursing care team. The nursing care team evaluated the patients and immediately notified an emergency physician. The team followed the acute stroke protocol of the Thammasat University Hospital (TUH). Laboratory in the protocol included blood glucose, complete blood count, and coagulogram. Two intravenous lines were obtained with 0.9% normal saline at 100 milliliters per hour. A neurologist on call was then notified. NIHSS was obtained by the emergency physician and the neurologist. The noncontrast CT brain was obtained as soon as possible. The team gathered all data and made decision for i.v. rt-PA administration by inclusion and exclusion criteria under the SFT protocol. The flow chart of SFT protocol of TUH is shown in Figure 1. The inclusion and exclusion criteria in SFT protocol of TUH were shown in Table 1. Once the decision to administrate i.v. rt-PA was made, 0.9 milligrams per kilogram (but not exceeding 90 milligrams) of rt-PA with 10 percent giving bolus and 90 percent intravenous dripping in one hour was promptly initiated. Vital signs were recorded every 15 minutes until the treatment was finished at the emergency department. Blood pressure was controlled below 185/110 mmHg before initiation and during continuous dripping of i.v. rt-PA. Then, the patients were transferred out to the stroke unit. The patients were under the care of multidisciplinary care team at stroke unit until they were discharged from the hospital. NIHSS was evaluated and CT brain was done at 24 hours after i.v. rt-PA administration.

#### 2.4.2. Experimental Group

**Figure 2:**
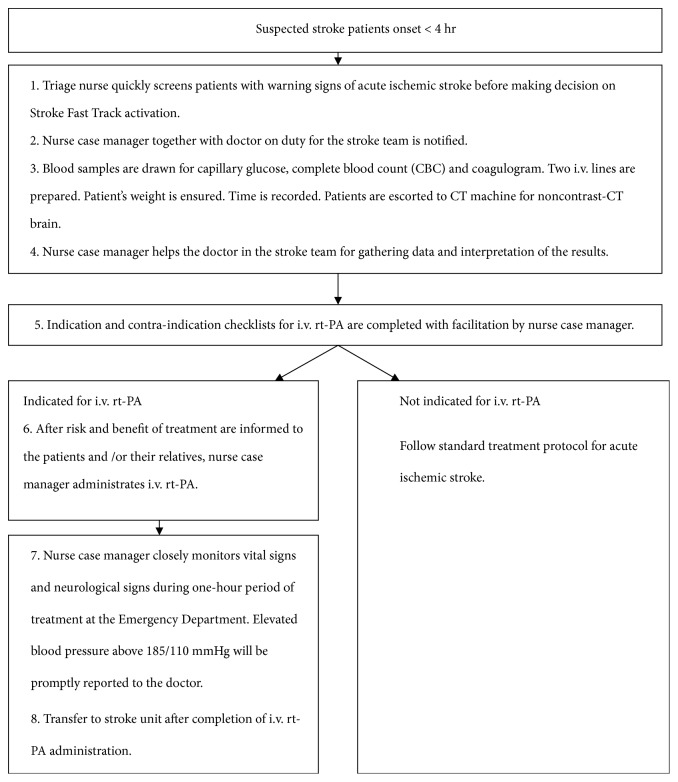
The schematic flow of nurse case manager for Stroke Fast Track.

The standard care was under responsibility of nurse case management and physicians. Nurse case management is the additional care for some specific conditions. The main purpose of nurse case management is to improve the quality of care in those specific conditions. The nurse, the so-called case manager, is responsible for nurse case management. The case manager has special knowledge to collect, assess, and analyze data regarding needs of those specific conditions. Therefore, case manager helps to identify problems of patients with specific conditions correctly and accurately. Case manager needs to have leadership in the management, including coordinating among related members of the team, managing technological and financial resources to provide effective and reasonable care to patients, conduct information management to report outcomes of the operation, and to negotiate with the treatment team and related persons, and having an outcome-based management to cover all aspects of patient care. The case manager has to protect the rights of patients and create appropriate alternative care for patients, including clear clinical roles, effective information management, good personality, good rapport among team members, and positive attitudes toward the work and the team. The acceptance of the nurses' roles of case managers in the team should reduce obstacles and conflicts leading to more effective patient care processes. The schematic flow of nurse case manager for Stroke Fast Track was showed in Figure 2.

### 2.5. Data Collection Procedure

When patients with potential stroke came in, the triage nurse activated SFT protocol and promptly alerted the nurse case manager. The nurse case manager conducted and played a major role in each step of SFT protocol. As the major role of SFT protocol conductor, the case manager would, for instance, ensure time for laboratory results, accelerate time to reach CT machine, facilitate CT brain interpretation, provide equipment for NIHSS, gather all the mandatory data for decision-making on i.v. rt-PA administration, help to inform patients and family about benefit and risk of i.v. rt-PA administration, and intensively monitor clinical and vital signs during thrombolytic treatment. The patients were escorted by the case manager while they were transferred out to the stroke unit. The case manager also became a part of multidisciplinary care team in the stroke unit.

### 2.6. Data Analysis

(1) Demographic data, comorbid risk factors, clinical presentations, the dispatch method, arrival time to the hospital, and duration from triage to initiation of thrombolytic treatment are analyzed with descriptive statistics including frequency, percentage, means, and standard deviations.

(2) The NIHSS at 24 hours after treatment between control and experimental groups is compared with ANCOVA by controlling age, comorbid risk factors, hospital arrival time, and the location of the infarct.

## 3. Results

Seventy-six patients were randomized into thirty-eight cases equally in each group. Most of the patients were male (70.5%), aged between sixty and seventy years (57%), and with primary school education (73.5%). The most common risk factor was hypertension (41.2%). The most common clinical presentation was hemiparesis (50.6%). Most of the patients were dispatched with private transportation (57%). Most of the patients and/or their relatives were aware of their stroke symptoms (52.1%). Baseline characteristics were shown in Table 2.

Time from triage to treatment in the experimental group was significantly faster than in the control group (mean = 39.02 and 59.37 minutes, respectively; p=.001) as shown in Table 3.

Initial NIHSS, which represented the severity of the stroke, before thrombolytic treatment was not significantly different between experimental and control group (p=.513). However, the NIHSS at 24 hours after thrombolytic treatment was significantly improved in the experimental group (p=.001), as shown in Table 4. No symptomatic intracranial hemorrhage (sICH) was detected by following up CT brain at 24 hours after treatment in both groups.

The nurse case management significantly affected the neurological recovery of patients with thrombolytic treatment (p=.001) when using age, risk factors, subtype of infarct, severity of stroke, and time from stroke symptom onset to the hospital as covariates, as shown in Table 5.

The NIHSS at 24 hours after treatment in the nurse case management group was significantly improved as compared with that of the control group (p=.001). Moreover, time from triage to treatment was significantly shorter in the nurse case management group as compared with that in the control group (p=.001). When age, risk factors, subtype of the infarct, NIHSS, and time from symptom onset to the treatment were used as covariates, the nurse case management significantly facilitated early neurological recovery in patients with acute ischemic stroke with thrombolytic treatment (p=.001).

## 4. Discussion

Our study demonstrates the early benefit of a nurse case management in acute stroke care. The benefit does not depend on comorbid risk factors, age, subtypes of stroke, time from onset to treatment, or severity of the stroke of patients (as shown in Table 5). Standard guidelines recommend that the average door-to-needle time for i.v. rt-PA should be less than 60 minutes [16, 17]. Average door-to-needle time is less than 60 minutes in both groups. It represents the standard of acute stroke care in our center and is still comparable with our previous report [10]. However, the earlier i.v. rt-PA administration leads to the better clinical outcomes [18, 19]. The door-to-needle time in nurse case management group is significantly shorter than that of the control group. This can be the main reason for good early outcomes.

For safety concerns, computerized tomography of the brain is routinely done 24 hours after i.v. rt-PA administration to detect intracranial hemorrhage as a standard protocol [20]. No symptomatic intracranial hemorrhage (sICH) is detected at 24 hours after onset in both groups. This result supports the safety use of i.v. rt-PA in patients with acute ischemic stroke [10, 21]. However, with the number needed to harm around 40 for sICH [22], the small sample size, such as our study, may underestimate the events.

We propose that the roles and managerial competencies of the nurse case manager to enhance acute stroke care should consist of some essential specialty. A nurse case manager needs to have clinical knowledge and ability to promptly and accurately collect, assess, and analyze the data [23]. Leadership in the management is important [24]. This includes coordinating among related members of the team, managing technological and financial resources to provide effective and reasonable care to patients, conduct information management to report outcomes of the operation and to negotiate with the treatment team and related persons, and having outcome-based management to cover all aspects of patient care [25]. A nurse case manager needs to protect the rights of patients and create appropriate alternatives for patients which include clear clinical roles, effective information management, good personality, good rapport among team members, and positive attitudes toward the work and the team so as to ensure acceptance of the nurses' roles of case managers and to reduce obstacles and conflicts, hence more effective patient care processes [26]. Nurse case management should be implemented in acute stroke care to ensure that patients received immediate treatment by the administration of rt-PA so as to promote their neurological recovery. The nurse case management should reduce door to rt-PA administration time. This nursing case management program should be implemented in healthcare settings. The nursing case management program improves the quality of care for acute ischemic stroke patients.

In conclusion, the nurse case management should be implemented in acute stroke system. Although the early benefit is demonstrated in our study, further studies are needed to ensure the long-term benefit and confirm its gain in patients with acute ischemic stroke.

## Figures and Tables

**Table 1:** Inclusion and exclusion criteria for i.v. rt-PA at Thammasat University Hospital.

**Inclusion criteria for i.v. rt-PA administration:**	
□ 1. Clinical diagnosis of acute stroke	
□ 2. Well-established onset less than 4.5 hours	
□ 3. No contra-indication for rt-PA administration as per exclusion criteria	
□ 4. Non-contrast CT (NCCT) brain: negative for hemorrhage	

**Exclusion criteria for i.v. rt-PA administration:**	
□ 1. Only minor stroke symptoms	
-Pure sensory symptoms	
-Ataxia only	
-Motor score < 1 on initial NIHSS	
□ 2. Major symptoms are recovered before initiation of i.v. rt-PA infusion.	
□ 3. Any evidence of hemorrhage in the initial NCCT.	
□ 4. Highly suspicious of SAH although the initial NCCT is negative.	
□ 5. Evidence of pregnancy	
□ 6. Laboratory values:	
-Platelet count < 100,000	
-INR > 1.7	
-Glucose < 50	
-Hb < 10	
□ 7. Major surgery or serious trauma within the last 14 days	
□ 8. Serious head trauma or intracranial surgery in the last 3 months	
□ 9. Intracranial neoplasm, arteriovenous malformation, or aneurysm	
□ 10. Active GI or GU bleeding or history of such bleeding within the last 21 days	
□ 11. Recent arterial puncture at a non-compressible site (i.e., subclavian)	
□ 12. Lumbar puncture within the last 7 days (including epidural/spinal anesthesia)	
□ 13. Persistent hypertension	
-Unable to lower blood pressure below 185/110 within time window of treatment	
-Too aggressive treatment required to reduce blood pressure below 185/110	
□ 14. History of major cerebral infarction within the past three months	
□ 15. History of intracranial hemorrhage considered to put the patient at increased risk of intracranial hemorrhage	
□ 16. Concurrent serious illness	
□ 17. Clinical diagnosis of myocardium infarction or aortic dissection	
□ 18. Clinical presentation suggestive of post-myocardial infarction pericarditis	
□ 19. Seizures at onset of acute stroke	
□ 20. History or clinical/lab evidence of acute drug abuse	
**Additional exclusion criteria for symptom onset during 3 to 4.5 hrs:**	
□ 1. Age > 80 years	
□ 2. Taking Warfarin regardless of INR	
□ 3. Initial NIHSS > 25	
□ 4. Diagnosed as diabetic mellitus with previous ischemic stroke	

i.v. rt-PA = intravenous recombinant tissue plasminogen activator; NCCT = noncontrast computer tomography; SAH = subarachnoid hemorrhage; NIHSS = National Institute of Health Stroke Scale; INR = international ratio; Hb = hemoglobin; GI = gastrointestinal; GU = genitourinary.

**Table 2:** Baseline characteristics.

Characteristics	Experimental group		Control group		p-value
	Number (38)	%	Number (38)	%	
Gender					

Male	30	78.95	24	63.16	.508
Female	8	21.05	14	36.84	

Age					

< 50	4	10.52	6	15.78	.598
50-59	10	26.32	10	26.32	
>60	24	63.16	22	57.9	

Risk factors					

Hypertension	29	76.32	29	76.32	.365
Dyslipidemia	17	44.74	17	44.74	
Diabetes	13	34.21	12	31.58	
Atrial fibrillation	12	31.58	12	31.58	
Previous TIA	1	2.63	0	0	
Smoking	15	39.47	12	31.58	

Clinical presentation					

Hemiparesis/paresthesia	35	92.1	37	97.37	.756
Speech disorders	20	52.63	22	57.89	
Facial weakness	14	36.84	14	36.84	
Vertigo/imbalance	1	2.63	0	0	

Subtype of infarct					

Lacunar infarct	19	50	19	50	1.0
Nonlacunar infarct	19	50	19	50	

Dispatch					

Self-dispatch	23	60.53	20	52.63	.549
Interhospital	15	39.47	18	47.37	
EMS	0	0	0	0	

TIA = transient ischemic attack and EMS = emergency medical service.

**Table 3:** The parameters of time from symptom onset to hospital, time from hospital to received rt-PA, and time from onset to received rt-PA were compared between experimental and control groups.

Sample characteristics	Experimental group (n=38)		Control group (n=38)		t-test
	**Number**	**Percentage (**%**)**	**Number**	**Percentage (**%**)**	
Time from symptom onset to hospital					.994

15 - 30 minutes	2	5.26	2	5.26	

31 - 60 minutes	6	15.78	2	5.26	

61 – 90 minutes	3	7.89	7	18.42	

91 - 120 minutes	6	15.78	11	28.94	

121 – 150 minutes	12	31.57	6	15.78	

151 – 180 minutes	6	15.78	4	10.52	

181 – 210 minutes	3	7.89	4	10.52	

Mean standard deviation	$\overset{-}{x}$=119.29	SD =49.00	$\overset{-}{x}$=113.00	SD =51.10	

Time from hospital to treatment					.001

0 – 30 minutes	1	3.33	16	42.11	

31 – 60 minutes	24	63.16	19	50	

61 – 90 minutes	11	36.67	2	5.26	

91 – 120 minutes	2	5.26	1	2.63	

Mean standard deviation	$\overset{-}{x}$=39.02	SD=0.68	$\overset{-}{x}$=59.30	SD =0.75	

Time from symptom onset to treatment					.013

61 – 90 minutes	2	5.26	2	5.26	

91 – 120 minutes	6	15.79	3	7.89	

121 – 150 minutes	5	13.16	4	10.53	

151 - 180 minutes	14	36.84	13	34.21	

181 - 240 minutes	9	23.68	10	26.32	

241- 270 minutes	2	5.26	6	15.79	

Mean standard deviation	$\overset{-}{x}$=162.79	SD=16	$\overset{-}{x}$=179	SD=16.5	

**Table 4:** Mean and SD of NIHSS in experimental and control groups before and after receiving i.v. rt-PA within 24 hours by independent sample *t*-test analysis.

Parameters	Experimental group (n=38)		Control group (n=38)		*t*-values (t)	
	**Mean**	**Standard deviation (SD)**	**Mean**	**Standard deviation (SD)**		**p-value ** **(P)**
NIHSS before patients received i.v. rt-PA	13.2105	6.22618	14.1842	6.68138	.657	.513

NIHSS after patients received i.v. rt-PA	6.6579	6.39373	12.3947	7.66720	3.542	.001

**Table 5:** Comparison of mean NIHSS at 24 hours after intravenous rt-PA between experimental group and control group by ANCOVA test [control variables which are not related to the experimental group but affected covariates include age, risk factors, subtype of infarct, severity of stroke, and time from stroke symptom onset to hospital (n=76)].

	df	Sum of square	Mean square	F	p-value
Covariates and the groups	1	616.235	616.235	25.484	.001

Risk factors	1	.238	.238	.011	.915

Age (years old)	1	12.954	12.954	.620	.434

Subtype of infarct	1	7.757	7.757	.371	.544

Time (minutes)	1	1743.261	1743.261	68.765	.461

Severity of stroke	1	1913.994	1913.994	83.232	.100

Error values	70	1461.833	20.883		

Total	76	11210.000			

## Data Availability

The data used to support the findings of this study are available from the corresponding author upon request.
